# B-Virus (*Cercopithecine herpesvirus* 1) Infection in Humans and Macaques: Potential for Zoonotic Disease

**DOI:** 10.3201/eid0902.020272

**Published:** 2003-02

**Authors:** Jennifer L. Huff, Peter A. Barry

**Affiliations:** * University of California, Davis, California, USA

**Keywords:** *Cercopithecine herpesvirus* 1, B virus, herpes B, monkey B virus, *Herpesvirus simiae*, alphaherpesvirus, zoonoses, primate, macaque, rhesus, herpesvirus B, synopsis

## Abstract

Nonhuman primates are widely used in biomedical research because of their genetic, anatomic, and physiologic similarities to humans. In this setting, human contact directly with macaques or with their tissues and fluids sometimes occurs. *Cercopithecine herpesvirus* 1 (B virus), an alphaherpesvirus endemic in Asian macaques, is closely related to herpes simplex virus (HSV). Most macaques carry B virus without overt signs of disease. However, zoonotic infection with B virus in humans usually results in fatal encephalomyelitis or severe neurologic impairment. Although the incidence of human infection with B virus is low, a death rate of >70% before the availability of antiviral therapy makes this virus a serious zoonotic threat. Knowledge of the clinical signs and risk factors for human B-virus disease allows early initiation of antiviral therapy and prevents severe disease or death.

Of the 35 herpesviruses identified in nonhuman primates, only *Cercopithecine herpesvirus* 1 (B virus) is known to be pathogenic for humans. Monkeys of the genus *Macaca*, which are widely used as animal models for biomedical research, naturally carry B virus. Infection in macaques is lifelong, with periodic, usually asymptomatic reactivation.

Approximately 40 cases of zoonotic B-virus infection have been reported. Considering the number of people in contact with macaques, this number of cases is quite low. However, the death rate for B-virus infection before the availability of antiviral therapy is >70%. Neurologic sequelae are common in survivors. Treatment with antiviral medication may decrease the death rate, but rapid diagnosis and initiation of therapy are essential in controlling the spread of the virus in the central nervous system and limiting neurologic sequelae.

## Discovery of B-Virus

The first documented case of human B-virus infection occurred in 1932 when a researcher (patient W.B.) was bitten on the hand by an apparently healthy rhesus macaque (*Macaca mulatta*) and died of progressive encephalomyelitis 15 days later. Two research groups obtained samples from patient W.B.: Gay and Holden and Sabin and Wright (*1*,*2*). Both groups demonstrated a similar disease progression in rabbits inoculated with nerve tissue from patient W.B. and characterized the agent as a herpesvirus. Neither group was able to produce disease in rhesus macaques, presumably because the monkeys were already naturally infected with what Sabin’s group named B virus (after patient W.B.).

The familiar term B virus will be used throughout this article. Many other accepted terms for this virus exist, including Herpesvirus simiae, herpes B, monkey B virus, and herpesvirus B. The International Committee on the Taxonomy of Viruses uses the name *Cercopithecine herpesvirus* 1 (family: *Herpesviridae*, subfamily: *Alphaherpesvirinae*, genus: *Simplexvirus*). This designation is based on virologic characteristics and serologic cross-reactivity with other members of the genus *Simplexvirus*, namely HSV type 1 (HSV-1), the causative agent of oral herpetic ulcers (cold sores) in humans and HSV type 2 (HSV-2), the agent of human genital herpes (*3*).

## Structure and Life Cycle

B virus is a large, double-stranded DNA virus with numerous open reading frames, some of which share approximately 79% amino acid sequence identity with HSV-1 and HSV-2 (*4*). The viral genome is G+C rich (75% G+C), the highest of any known herpesvirus (*4*). The B-virus genome is only partially sequenced, but thus far, is colinear with that of HSV (*5*). Electron micrograph studies of B virus show a typical herpesvirus structure (*6*), including an electron dense core with viral DNA inside an icosapentahedral capsid surrounded by an amorphous tegument protein layer and a lipid envelope studded with viral glycoproteins. Glycoproteins on the viral envelope mediate attachment to and entry into the host cell. For HSV, 11 glycoproteins are known (gB–gM), and another is predicted (gN). Of these, nine have been identified in B virus (*5*).

In general, alphaherpesviruses infect mucosal epithelia followed by one or more rounds of replication in epithelial cells. B virus likely replicates with three consecutive rounds of transcription (the α, β, and γ genes), as has been established for HSV. The infected cells are lysed, releasing virus to spread to other cells and sensory nerve endings, although direct entry into neurons without replication can occur (*3*). Virus can also spread from cell to cell without contacting the extracellular environment. Spread of the virus to and from the nerve ganglia occurs by axonal transport, which has been demonstrated for B virus in experimentally infected mice (*7*). The virus establishes latency in the nerve ganglia. Latency is characterized by a lack of viral replication and limited viral transcription. Periodic reactivation from latency delivers the virus to mucosal epithelial cells, where it replicates; infectious virus is released from the mucosal epithelium. A heavy viral load in the ganglia may increase the frequency of reactivation and shedding (*8*). Recent findings from the study of primary and recurrent HSV-2 infection indicate that most episodes of recurrent viral shedding are asymptomatic. B-virus shedding in macaques also appears to be primarily asymptomatic (*9*–*11*).

## B-Virus Infection in Macaques

B-virus infection has been reported most commonly in the rhesus and cynomolgus macaque (*M. facicularis*), two species used extensively in biomedical research. B virus has also been isolated from the stumptail (*M. artoides*), pig-tailed (*M. nemestrina*), Japanese (*M. fuscata*), bonnet (*M. radiata*), and Taiwan (*M. cyclopis*) macaque (*12*). Sequence comparisons and restriction fragment length polymorphism analysis of viral genomes have demonstrated strain differences between B-virus isolates from different macaque species (*13*). Research suggests that B virus from rhesus macaques may be more pathogenic for humans than B virus from other macaque species (*13*). Where the species of macaque is noted, cases of human B-virus infection have all been associated with direct or indirect exposure specifically to rhesus macaques (*14*–*19*). The ability of nonrhesus strains of B virus to infect humans is not well understood.

Little is known about the biology of B virus in its natural host. Infection is usually acquired at sexual maturity (2–4 years of age for rhesus macaques). As seen in humans with HSV, B-virus seropositivity increases with population age; seropositivity rates of 80% to 100% occur among most adult captive macaque populations (*10*,*20*,*21*). Oral herpetic lesions such as gingivostomatitis, oral and lingual ulcers, and conjunctivitis have been described, but are usually associated with immunosuppression or stress attributable to recent importation or crowded housing conditions (*12*,*22*,*23*). Genital lesions have not been observed in macaques, although genital infection has been demonstrated by polymerase chain reaction (PCR) (*9*), virus isolation from the genital mucosa (*10*,*11*), and culture of the sacral ganglia (*11*). In general, macaques remain asymptomatic, and identification of oral herpetic lesions is sufficient grounds for euthanasia of the affected animal. The infrequent cases of disseminated B-virus disease in macaques are most often associated with immunosuppression, caused by either chemotherapy or concurrent infection as with simian type D virus (*22*). Although severe HSV disease is commonly observed in humans co-infected with HIV, no cases of B-virus disease associated with simian immunodeficiency virus infection in macaques have been reported (*24*).

Relatively few studies have surveyed macaques for B-virus shedding, and detection of B virus by culture is rare. Most cases of B-virus detection in asymptomatic macaques by culture or PCR are associated with breeding season stress (*9*,*10*), immunosuppression (*25*), or primary infection (*10*,*11*). The true frequency of B-virus shedding in macaque populations is not known but is likely to be low.

Most cases of human B-virus infection have been associated with apparently healthy macaques (i.e., no obvious herpetic lesions), which indicates asymptomatic shedding of the virus. Lack of clinical signs of recurrent infection makes identification of shedding animals difficult. People working with these animals should consider every animal a potential source of B virus and use proper protective equipment and care when handling them (*21*,*26*–*28*).

## Human B-Virus Infection

Most cases of human B-virus infection have involved direct contact with macaques, such as a bite, scratch, or mucosal contact with body fluid or tissue (*12*,*14*–*16*,*19*,*27*,*28*). Indirect contact, such as injury from a contaminated fomite (e.g., needle puncture or cage scratch), has also resulted in human infection. Human-to-human transmission has been documented in one case (*15*); however, further investigation has indicated that the risk for secondary transmission is low (*18*).

Human B-virus disease generally occurs within 1 month of exposure (*21*), commonly with an incubation period of a few days to a week. The development and progression of disease depend on the site of exposure and the amount of virus inoculated. Vesicular lesions have not been consistently found at the site of exposure (*12*,*14*–*19*). Disease often starts with general influenzalike symptoms of fever, muscle aches, fatigue, and headache (*12*,*14*). Other variable symptoms include lymphadenitis and lymphangitis, nausea and vomiting, abdominal pain, and hiccups (*12*,*14*). Neurologic signs develop when the virus spreads to the central nervous system and vary with the part of the brain or spinal cord affected. Hyperesthesias, ataxia, diplopia, agitation, and ascending flaccid paralysis have been described after virus spread to the brain (*12*,*14*–*19*). Virus spread to the central nervous system is an ominous sign; even with antiviral therapy and supportive care, most patients die, and those who survive often have serious neurologic sequelae. Deaths are often attributed to respiratory failure associated with ascending paralysis.

The possibility of asymptomatic or mild B-virus infection in humans has been suggested (*2*,*29*). A carefully controlled study of B-virus antibodies in persons with macaque contact and controls without contact showed no evidence of asymptomatic human infection or a carrier state for B virus (*29*). Although HSV antibodies can neutralize the virus in vitro*,* antibody titers to HSV are not protective in human cases of B-virus exposure or infection (*21*,*29*) and can confound diagnostic testing because of cross-reactivity. Asymptomatic human infection with B virus appears exceedingly rare if it occurs at all.

With the discovery of simian immunodeficiency virus and its identification as a model for HIV infection, the number of macaques used in research has increased, as has the number of human B-virus cases. Guidelines for reducing and controlling exposure were first published in 1987 (*26*) by a group of veterinarians, physicians, and research scientists called the B Virus Working Group. Guidelines were again published in 1995 (*21*) by a new B Virus Working Group to include new information and provide protocols for handling exposures. Further recommendations were made in 1998 to emphasize the need for limiting mucosal exposure to potential sources of B virus (*19*,*28*). New guidelines by another B Virus Working Group have recently been published (*30*).

## Treatment of B-Virus Infection in Humans

The 2002 B Virus Working Group guidelines address issues to be considered in cases of possible exposure to or infection with the virus (*30*) and reflect consensus of opinion at the time the guidelines were written. In cases of exposure, an established and frequently updated protocol should be used based on these guidelines and on current literature regarding human cases of B-virus infection. Additional information and contacts are available from: URL: http://www.cdc.gov/niosh/hid5.html, http://www.haz-map.com/Macaque.htm.

According to the guidelines, the most important action in a case of potential exposure to B virus is to rapidly and thoroughly cleanse the wound or exposure site. HSV can enter sensory nerve endings within 5 minutes of exposure, and B virus is likely to infect just as rapidly. Bite wounds, scratches, or puncture wounds of nonmucosal surfaces should be cleansed with soap or detergent for at least 15 min (*30*). The time spent mechanically cleansing the area is more important than the type of cleansing solution used. Mucosal surfaces should be rinsed with sterile saline or running water for 15 min. Immediate cleansing or rinsing can inactivate and wash away virus present in the exposure site. After immediately cleansing the wound or exposure area, the person should seek medical attention, specifically from a physician identified in the facility’s protocol as someone familiar with treating these B-virus exposure cases.

A physician with a patient who has potentially been exposed to B virus faces a conundrum. Before onset of neurologic symptoms, antiviral therapy is successful. However, few cases of potential exposure lead to infection. Prophylactic treatment is unnecessary in most cases of potential exposure because treatment can confound diagnostic testing by interfering with the humoral immune response (*21*). However, the 2002 B Virus Working Group viewed prophylactic treatment more favorably in light of the efficacy of postexposure prophylaxis for nosocomial HIV exposure and the availability of new antiherpes drugs, such as valacyclovir, that achieve higher serum levels with a more reasonable dosing schedule (*30*). Although severity of injury may prompt use of antiviral therapy, the amount of inoculated virus determines if infection is likely to occur. In some cases, minor scratches or needle-sticks have transmitted B virus, while bites with severe tissue laceration have healed without infection. The primary factor to consider is whether cleansing (or rinsing, if it is a mucosal surface) was initiated immediately and performed for the recommended 15 min (*21*). Inadequate cleansing of the wound or exposed area in a timely manner could warrant prophylactic antiviral therapy. Other indications for immediate initiation of antiviral treatment include the identification of herpetic lesions in the source animal, injuries involving the head or neck, and mucosal exposure to macaque fluid. Because of the prevalence of asymptomatic B-virus shedding in macaques, the clinical appearance of the monkey involved (if the animal is identified) may not be helpful in evaluating the possibility of transmission.

In addition to working closely with a physician trained to handle cases of B-virus exposure and infection, taking samples from the exposed person and the source animal is important for virus culture and serologic testing. A list of recommended swabs for virus culture and serum samples is available from: URL: http://www.gsu.edu/~wwwvir/index.html.

## Detection of B-Virus

Early suspicion and rapid diagnosis of B-virus infection are critical to the control of human infection. The extreme cross-reactivity of primate alphaherpesviruses has required the development of diagnostic methods that can differentiate between HSV and B-virus infection. Despite the inherent risk for exposure, direct culture of B virus has been the standard for diagnosis of infection. Culture of B virus requires a special containment facility since the virus is a biosafety level 4 pathogen (*31*). Serologic methods for the detection of B-virus infection have also involved propagation of the virus in tissue culture to produce antigen. However, the substitution of related antigens appears to work well for serologic tests. The most promising of these antigens is herpesvirus papio 2, an alphaherpesvirus of baboons that is as closely related to B virus as HSV-1 and HSV-2 are to each other (*32*,*33*). Serologic methods are useful only for retrospective analysis, not for therapeutic decisions, which need to be made rapidly in cases of potential human infection.

More recently, PCR methods have allowed direct demonstration of B-virus infection without the risk of working with virus cultures (*9*,*34*,*35*). PCR methods have been hampered by the close genetic relationship between primate alphaherpesviruses; many require post-PCR techniques to definitively differentiate between HSV and B virus. To specifically detect B virus, we developed a method using quantitative real-time PCR, whose potential application for human clinical samples in cases of exposure warrants further study (*9*). Samples to be tested by PCR may contain B virus and must be handled accordingly (*31*).

## B-Virus Outside the Research Setting

The cases of human B-virus infection that have been described have all occurred in relation to contact with macaques in a biomedical research setting. However, this setting is not the only one in which humans have contact with macaques. The Woburn Safari Park in the U.K. recently culled all B-virus–positive macaques from its facility (*36*). No cases of human infection have been documented despite contact between macaques and humans driving through the park, but the risk perceived by this situation warranted the action. B virus is also prevalent in free-ranging macaques native to Southeast Asia (*12*,*37*). A recent survey of workers at a Balinese Hindu temple that is a refuge for free-ranging macaques and a tourist attraction showed that contact between humans and macaques sufficient to transmit B virus commonly occurred. A serosurvey of 38 macaques in the area showed that 31 (81.6%) were B-virus seropositive. No cases consistent with B-virus disease in humans have been described in this area of Bali or in other areas of Southeast Asia where humans are in contact with free-ranging macaques. However, in cases of encephalitis, B virus may not be considered.

In other situations, particularly when potentially seropositive macaques have been domesticated as pets, opportunities for exposure to B virus are frequent. One report documented many instances of potential exposures from bites, scratches, food sharing, close physical contact, and even shared chewing gum (*38*). This study also found that children were three times more likely than adults to be bitten by pet macaques. Although the number of macaques kept as pets is probably small, the risk of B-virus infection is increased because of the lack of precautions and the extent of contact between monkey and owner. The risk of B-virus infection is low, but the risk for death is high.

## Specific Pathogen-Free Colony Development

In 1989, the National Institutes of Health’s National Center for Research Resources started funding specific pathogen-free macaque colony development. The timing and local nature of B-virus reactivation and shedding make detecting infection in an animal difficult. Therefore, serologic methods are used to screen and monitor animals for consideration as pathogen-free. Numerous negative serologic results are necessary to determine a macaque’s B-virus status. Although specific pathogen-free status reduces the likelihood of infection, this status does not eliminate the risk for infection entirely. Full protective equipment should be used for working with all macaques regardless of their pathogen status. While population numbers in these specific pathogen-free colonies are increasing, the demand for pathogen-free animals will continue to exceed the supply for some time (*24*).

## B-Virus Vaccine Development

While antiviral therapy has substantially improved the survival rate for human B-virus infection, fatal cases still occur (*19*,*28*). The ability of the virus to modulate and evade the immune response has stymied vaccine development for most herpesvirus infections. A vaccine for use in rhesus macaques could reduce transmission of the virus and, over time, reduce the prevalence of infection in captive macaque populations. Given the lack of an effective vaccine for HSV after years of research effort and clinical trials, development of a B-virus vaccine presents a challenge.

A formalin-inactivated B-virus vaccine was developed and tested in the 1960s (*39*). Although this vaccine did induce an antibody response, antibody titers were low, and frequent boosters (every 3 months) were required (*39*). Recently, successful use of a vaccinia vector to deliver the gD gene of B virus was demonstrated in rabbits with protection of 10 (91%) of 11 animals from B-virus challenge (*40*). A DNA vaccine against B virus has also recently been described (*41*). Glycoprotein B of B virus delivered in a plasmid vector induced a humoral response in both mice and rhesus macaques. Although no challenge experiments were performed in monkeys, an anamnestic-like response upon boosting was noted. While the ability of a B-virus antibody response to protect from infection is not known, studies of HSV suggest that an antibody response alone is not protective. Both the vaccinia and DNA vaccine approaches described above are likely to induce cellular immunity to B virus, although the cellular response was not studied by either group (*40*,*41*). As clinical trials of candidate HSV vaccines progress, the development of a B-virus vaccine for use in macaques or humans at risk for exposure should be considered.

## Conclusion

The potential for fatal human infection with B virus is a constant concern because frequent exposures occur to humans in the course of caring for and using macaques in a research setting. Personal protective equipment and safe handling procedures have limited the incidence of human disease. However, little is known about the biology of B virus in the natural macaque host. A clear understanding of the real risk for B-virus shedding in its natural host will help identify opportunities to prevent or limit zoonotic B-virus disease.
